# Immunization of Mice by BCG Formulated HCV Core Protein Elicited Higher Th1-Oriented Responses Compared to Pluronic-F127 Copolymer

**DOI:** 10.5812/hepatmon.14178

**Published:** 2013-10-23

**Authors:** Maryam Yazdanian, Arash Memarnejadian, Mehdi Mahdavi, Seyed Mehdi Sadat, Fatemeh Motevali, Rouhollah Vahabpour, Hossein Khanahmad, Seyed Davar Siadat, Mohammad Reza Aghasadeghi, Farzin Roohvand

**Affiliations:** 1 Hepatitis and AIDS Department, Pasteur Institute of Iran, Tehran, IR Iran; 2 Virology Department, Pasteur Institute of Iran, Tehran, IR Iran; 3 BCG Research Center, Karaj Research and Production Complex, Pasteur Institute of Iran, Karaj, IR Iran; 4 Microbiology Department, Pasteur Institute of Iran, Tehran, IR Iran

**Keywords:** HCV, Adjuvant, Bacillus Calmette-Guerin (BCG), PluronicF127

## Abstract

**Background:**

A supreme vaccine for Hepatitis C virus (HCV) infection should elicit strong Th1-oriented cellular responses. In the absence of a Th1-specific adjuvant, immunizations by protein antigens generally induce Th2-type and weak cellular responses.

**Objectives:**

To evaluate the adjuvant effect of BCG in comparison with nonionic copolymer-Pluronic F127 (F127) as a classic adjuvant in the formulation of HCV core protein (HCVcp) as a candidate vaccine for induction of Th1 immune responses.

**Materials and Methods:**

Expression of N-terminally His-Tagged HCVcp (1-122) by pIVEX2.4a-core vector harboring the corresponding gene under the control of arabinose-inducible (*araBAD*) promoter was achieved in BL21-AI strain of *E.coli* and purified through application of nitrilotriacetic acid (Ni-NTA) chromatography. Mice were immunized subcutaneously (s.c.) in base of the tail with 100 μl of immunogen (F127+HCVcp or BCG+HCVcp; 5 μgHCVcp/mouse/dose) or control formulations (PBS, BCG, F127) at weeks 0, 3, 6. Total and subtypes of IgG, as well as cellular immune responses (Proliferation, *In vivo* CTL and IFN-γ/IL-4 ELISpot assays against a strong and dominant H2-d restricted, CD8+-epitopic peptide, core 39-48; RRGPRLGVRA of HCVcp) were compared in each group of immunized animals.

**Results:**

Expression and purification of core protein around the expected size (21 kDa) was confirmed by Western blotting. The HCVcp + BCG vaccinated mice showed significantly higher lymphocyte proliferation and IFN-γ production but lower levels of cell lysis (45% versus 62% in CTL assay) than the HCVcp+F127 immunized animals. “Besides, total anti-core IgG and IgG1 levels were significantly higher in HCVcp + F127 immunized mice as compared to HCVcp + BCG vaccinated animals, indicating relatively higher efficacy of F127 for the stimulation of humoral and Th2-oriented immune responses”.

**Conclusions:**

Results showed that HCVcp + BCG induced a moderate CTL and mixed Th1/Th2 immune responses with higher levels of cell proliferation and IFN-γ secretion, indicating that BCG may have a better outcome when formulated in HCVcp-based subunit vaccines.

## 1. Background

Infection with hepatitis C virus (HCV) is the major cause of liver disease such as fibrosis and cirrhosis (1). Approximately, 3% of the world’s population (with addition of 3-4 million annually) is infected with HCV. Despite these accelerating rates, no vaccine against this viral infection has been approved to date (2). High genetic heterogeneity of HCV (manifested by presence of at least six major genotypes) and induction of antibody and CTL escape mutants were among reasons hampering progress in HCV vaccine development (3). However, advances in understanding the correlates of protective immunity in HCV infection and vaccine formulations predict availability of HCV vaccines, in the near future (3, 4). Indeed, recent findings indicated that an appropriate HCV vaccine should elicit broad, cross protective and strong immunity against HCV conserved epitopes (mainly Th1-oriented cellular responses) manifested by high levels of proliferation rates, CTLs and interferon secretion (5, 6).

HCV genome contains a single-stranded positive-sense RNA that encodes for three structural proteins (core, and envelope proteins E1 and E2) and six nonstructural (NS) proteins (NS2, NS3, NS4A, NS4B, NS5A and NS5B) (3). Among HCV proteins, NS3 and particularly the core protein (HCVcp) represent the most conserved and protective T-cell epitopes between various HCV genotypes, a characteristic of high demand for HCV vaccine formulations (3, 7) which has even resulted in employment of isolated HCVcp-T cell epitopes in the context of a number of HCV multi-epitopic vaccines (8-10). Beside production of the HCV capsid protein, the nucleotide sequence of HCVcp encodes for other so called Core+1 or ARFP proteins through mechanisms such as ribosomal-frame shifts (11) or internal translation initiation (12). Immune responses against these recently identified HCVcp-derived proteins might have also potential implications in HCV vaccine formulations (3). However, prior studies have indicated that the C-terminal hydrophobic region of HCVcp negatively interferes with both innate and acquired immune responses through several immune-suppressive mechanisms (3, 13, 14). These preceding findings pointed to the inclusion of the hydrophilic region of HCVcp (encompassing the first 120 N-terminal residues) which contains both conserved T-cell epitopes (3, 15) and nuclear localization signals (NLS) (16) for formulation of various HCV vaccine candidates (15, 17-20).

Invention of subunit vaccines by formulation of the recombinant antigen(s) in adjuvant systems (21) clearly revolutionized the history of vaccines by introduction of the vaccine against hepatitis B virus infection (22). In fact, the earliest attempts on the development of candidate HCV vaccines also consisted of recombinant HCV-E1 or E1/E2 proteins formulated in alum or MF29 adjuvants respectively (reviewed in (4)). However, in the absence of a Th1-specific adjuvant, immunizations by protein antigens generally induce Th2-type and weak cellular responses (23). Since, the ultimate outcome of immune responses for combination of each protein and adjuvant may differ (24), formulation of HCVcp with different adjuvant systems (such as; montanides, ISCOMATRIX, Plouronic-F127) were studied [reviewed in (4)].

Recent advances in application of specific ligands of co-stimulatory molecules (25) and Toll-like receptors (TLRs) (26) as novel adjuvant systems for direct activation of T cells has recalled for possible application of live organisms like Bacillus Calmette-Guerin (BCG) as a class of immune potentiators. BCG is a well-known and the most widely used vaccine against tuberculosis (27) that acts directly on the immune system to augment cell-mediated and Th1-biased responses by several mechanisms such as; stimulating TLRs (28, 29), activating CTLs (30-32) and increasing the level of IFN-γ secretion (33, 34). These characteristics make BCG clearly an ideal adjuvant to provoke the cellular arm of the immune system for vaccination against other infections. In this context, BCG has already been found to have some roles as a human-compatible adjuvant in vaccination and immunotherapy trials against leishmaniasis (35-37)and to protect against schistosoma (38) and trypanosome (39) parasite infections. Although construction of recombinant BCG for expression of the CTL epitope of HCV NS5a protein (40) and a multi-epitopic HCV construct (41) and their application in murine immunization studies has been recently reported, but to our knowledge, no prior investigation has addressed any immunization studies on formulation of HCV proteins with BCG as an adjuvant system.

## 2. Objectives

To evaluate the adjuvant effect of BCG in formulation of HCVcp protein as a candidate vaccine in provoking the desired immune responses compared to Plouronic-F127 copolymer (F127), as a well-known human-compatible adjuvant.

## 3. Materials and Methods

### 3.1. Proteins and Peptides

The pIVEX2.4a-core plasmid encoding the HCVcp (amino acids 2-122) was used for the expression of N-terminally 6xHis-tagged HCVcp protein in BL21-AI strain of *E.coli* as previously described (42, 43). The protein was purified through application of nitrilotriacetic acid ( Ni-NTA) chromatography and further confirmed by western-blotting, based on the earlier reported methods (15, 43). Concentration of the HCVcp was determined by the BCA protein assay (Pierce; USA) and the endotoxin level was quantified by QCL-1000 Chromogenic Limulus amoebocyte lysate test (BioWhittaker), according to the manufacturer protocols. The C39 peptide corresponding to HCVcp residues 39-48 (RRGPRLGVRA) which is a strong and dominant H2-d restricted, CD8+-epitopic peptide (14, 15, 44) was synthesized with 95% purity (BIOMATIK Co. Canada) and used for analyses of all cellular responses throughout this study.

### 3.2. Immunogen formulations

BCG 1173-P2 Pasteur strain (Pasteur Institute, Iran) was diluted in phosphate-buffered saline (PBS) and mixed with purified HCVcp prior to injection to an administrable dose of 5 × 10^4^ CFU/mouse/dose(34). Pluronic F127 stock solution (Sigma, USA) was prepared at 16% (v/v) in PBS and mixed at 1:1 volume ratio with purified HCVcp protein as previously described (15). The administered dose of HCVcp was 5 μg/mouse/dose for all immunogen formulations.

### 3.3. Protocols of Immunization and Bleeding

Female BALB/c (H2d) mice (6–8 weeks old-average 20g of weight) were housed in approved animal-care facilities and were handled according to national animal care ethics. Five groups with 8 mice in each group were designated based on the received immunogen as PBS, BCG, HCVcp+BCG, F127 and HCVcp+F127 (Table 1). Mice were immunized subcutaneously (s.c.) at the base of the tail with 100 μL (total volume) of immunogens at weeks 0, 3, 6 and were bled through the retro-orbital plexus/sinus before each injection and three weeks after the last injection. Samples were centrifuged and collected sera were preserved at −70C. Control groups were injected with 100 μl of either PBS, or F127 or BCG formulation alone (without any HCVcp; Table 1).

**Table 1.** The Calculated Ratios of IgG2a/IgG1 Antibody Subclasses and IFNγ/IL-4 Cytokines in Immunized Mice Groups. Indicated Ratios for HCVcp + BCG Immunogen Formulation Compared to That of HCVcp + F127 Suggested Relatively Higher Th1-Oriented Immune Responses for the Preceding Formulation

Groups of Immunized Mice	IgG2a /IgG1 Ratio	IFNγ/IL-4 Ratio
**Saline**	NA^a^	NA
**BCG ^a^**	NA	NA
**HCVcp ^a^ + BCG**	0.36	8.25
**F127**	NA	NA
**HCVcp + F127**	0.22	2.45

^a^ Abbreviations: BCG, bacillus calmette-guerin; HCVcp, HCV core protein; NA, Not Applicable

### 3.4. Anti-HCVcp Antibody Assay

An indirect ELISA was developed to measure anti-HCVcp Abs in murine sera as previously described (15). To determine the antibody subclasses, mice sera were used at 1:10,000 dilutions. Here, biotinylated antibodies against mouse IgG1 or IgG2a (Sigma, USA, 1:1000 dilution), streptavidin-HRP conjugate (Sigma, USA, 1:3000 dilution) and consequently TMB substrate were used for detection. In both experiments absorbance was read at 450 nm.

### 3.5. Splenocyte Isolation

Three weeks after the last immunization, spleens of the individual mice were smashed into a cell homogenizer, washed with 2% fetal bovine serum (FBS)-PBS and treated with 0.83% NH4Cl solution for 6 min to lyse the erythrocytes. Washed spleen cells were resuspended in complete RPMI-1640 medium (Gibco, Germany) containing 10% FBS, 2 mM L-glutamine, 100 mg/mL streptomycin and 100 U/mL penicillin (all from Biosera, UK) and eventually used for cellular immune assays.

### 3.6. Cytokine ELISpot Assay

Enzyme-linked immune-spot (ELISpot) assay was used for ex vivo enumeration of the number of specific cells that were capable of secreting IFNγ and IL4 cytokines according to the manufacturer’s (Mabtech, Sweden) manual. In brief, splenocytes (3 × 105 cells/well) were plated in triplicate onto either anti-IFNγ or IL4 -coated 96-well plates and stimulated for 48 hrs with C39 epitopic peptide (20 μM). After cell removal, washing steps, treatment with enzyme-labeled secondary antibodies (against either IFNγ or IL4), and finally addition of chromogenic substrate spot forming cells (SFCs) were counted using a dissection stereoscope (Nikon, Japan). Wells containing phytohaemagglutinin (PHA, 2.5 µg/mL) served as positive control and cells cultured in the absence of C39 peptide served to find the background spots.

### 3.7. Proliferation Assay

splenocytes were seeded in triplicate wells of 96-well plates (1.5×105 cells/well) and incubated in the presence of stimulating peptide (C39 peptide, 20 µM) for 96 hrs at 37 C°. Proliferation rate of the specific cells was measured using the non-radioactive colorimetric method of “Cell Proliferation ELISA BrdU Kit” (Roche, Germany), according to manufacturer’s protocol. Briefly, incubated cells were pulsed with 100 mM 5-bromo-2 deoxyuridine (BrdU) labeling solution for 24 hrs at 37 C°, fixed and incubated for 120 min with HRP-conjugated mouse anti-BrdU-POD monoclonal Ab (1:100 dilution). After washing steps, addition of substrate solution (100 μL/well) for 30 min led to the color appearance, which was stopped by 1 M H2SO4. The absorbance was measured at 450 nm with a reference wavelength at 690 nm. Results were expressed as a stimulation index (SI), which was described by dividing absorbance of peptide stimulated to non-stimulated cells. Wells containing PHA served as a positive control.

### 3.8. In vivo CTL Assay

Detection of *in vivo* cytotoxic T lymphocytes (CTL) was performed according to a previously described method (45) with minor modifications. Splenocytes from naïve mice (6×107 cells/mL) were divided into two populations, one of which was pulsed with C39 peptide (10 µM) for 120 min at 37C. Cells were then washed and labeled with Carboxy Fluorescein diacetate, Succinimidyl Ester (CFSE) dye (Molecular Probes, USA) for 15 min at 37 C°. Peptide-pulsed and non-pulsed cells were respectively labeled with 5 µM (CFSE-high) and 0.5 µM (CFSE-low) of CFSE dye. Cells were then washed once in PBS–2% FBS, twice in PBS and equally mixed prior to intravenous injection into recipient mice. Twenty hrs after transfer, cells were recovered from the spleen of recipients and the relative proportion of CFSE high and CFSE low cells was determined by flow cytometry using a PAS instrument and the Flow Max software (Partec, Germany). Percent of specific lysis was calculated by the following formula: [1−(r unprimed/r primed)] × 100, where r = %CFSE-low/%CFSE-high for each mouse.

### 3.9. Statistical Analysis

Prism-4 software (Graph Pad, USA) was used for the data handling and statistical analysis was carried out using Mann–Whitney non-parametric and ANOVA tests. Statistical significance was set at P ≤ 0.05.

## 4. Results

### 4.1. HCVcp Production

**Figure 1.**
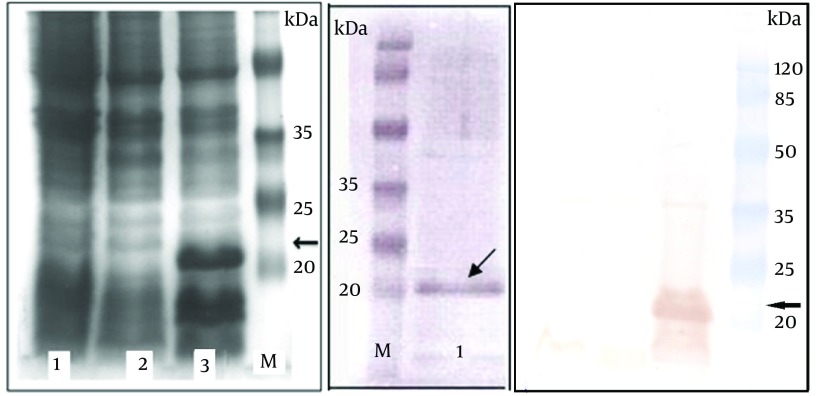
SDS- PAGE and western blot analysis of expressed HCVcp in *E.coli *-BL21AI (A) SDS–PAGE and (C) Western blot. Lane 1: crude untransformed bacterial extract. Lane 2: crude bacterial extract before induction by arabinose. Lane 3: crude bacterial lysate 4 h after induction. (B) Shows the SDS-PAGE analysis of HCVcp protein band purified on Ni-NTA column (lane 1). Lane M: protein MW marker in kDa. The arrows indicate the protein band with expected size of 21 kDa. The faint band over 35 kDa might be related to the dimer of the HCVcp, as previously suggested (15)

As shown in Figure 1 A, the coomassie blue-stained SDS–PAGE analysis of the bacterial lysates indicated expression of a protein band with the expected molecular weight of 21 kDa corresponding to 6xHis-tagged HCVcp. Densitometry analysis of the Ni-NTA chromatography-based purified protein bands in SDS-PAGE (Figure 1 B) demonstrated over 85% purity for HCVcp. Quantification of the endotoxin levels indicated less than 25 endotoxin units per 50 µg of the purified protein which was proper for the final aim of the immunization (Results not shown). Accordingly, western blot analysis by anti-HCV core monoclonal antibody recognizing amino acids 21-40 (Alexis Biochemical, UK) also confirmed the proper expression of the protein within the crude bacterial lysate (and the purified protein) and further indicated the proper antigenicity of the HCVcp protein purified by affinity chromatography (Figure 1 C).

### 4.2. BCG and F127 Adjuvant Effects on the Induction of HCVcp-Specific Humoral Response

**Figure 2.**
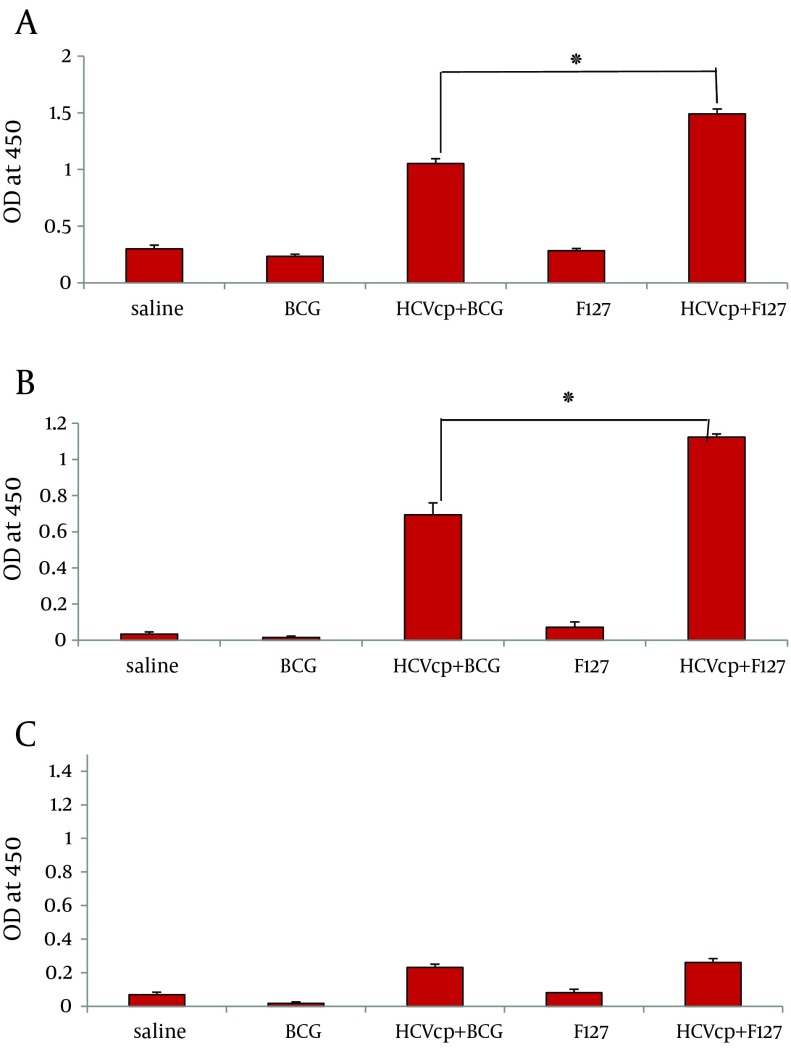
Analysis of humoral responses, (A) total IgG, (B) IgG1 and (C) IgG2a antibody levels in mice immunized with HCVcp in different formulations by ELISA. In control groups HCVcp is replaced by Normal Saline. Each formulation is written on the horizontal axis of diagrams (see text for detailed procedure). Data shows antibody titers in three independent experiments. Data are expressed as means ± standard errors per group and “*” (star labeled figure) indicates the significant differences (P < 0.05) according to the Mann–Whitney non-parametric test.

As depicted in Figure 2, sera from negative control groups that received either normal saline (mock) or sole adjuvants did not show any specific reactivity to HCVcp. By contrast, animals vaccinated with the adjuvant-formulated HCVcp demonstrated specific IgG antibodies 3 weeks after the last immunization. However, the level of total anti-core IgG raised in case of immunization with HCVcp+ F127 was significantly higher than that of the HCVcp + BCG. Besides, analyses of IgG1 and IgG2a subclasses as the representatives of Th2 and Th1 responses was performed (46). As shown in Figure 2 B, HCVcp+F127 formulation resulted in a considerable enhancement of IgG1 production compared to that of HCVcp + BCG (P < 0.05). Although assessment of the IgG2a subtypes, showed no significant difference between the two HCVcp groups of immunized mice (Figure 2 C) yet as could be anticipated, the calculated ratio of IgG2a/IgG1 (Table 1) clearly indicated a relatively higher Th1-oriented immune response for HCVcp + BCG immunogen formulation compared to that of HCVcp + F127.

### 4.3. BCG and F127 Adjuvant Effects on the Induction of HCVcp-Specific Cellular Response

**Figure 3.**
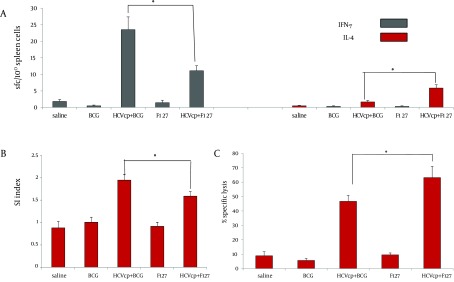
Type and Magnitude of Cellular Responses Induced in HCVcp-Adjuvanted Immunized Mice, (A) Enzyme-linked-immunospots assay for detection of class I-binding C39 peptide specific IFN-γ and IL-4-releasing T cells; results are shown as the numbers of spot-forming-cells (SFCs) per 106 splenocytes (3 weeks after the last immunization in BALB/c mice). Mice were immunized in two different regimens with HCVcp either formulated in BCG or F127 as adjuvant. Results indicated the significant differences between different regimens of immunization and a higher degree of IFN-γ and IL-4-releasing T cells in case of HCVcp+BCG and HCVcp+F127 formulations, respectively. (B) Assessment of mice spleen T lymphocyte proliferation using the BrdU ELISA; Proliferation assay of BCG-core and F127-core vaccinated mice splenocyts and control groups showed significant difference of stimulation index (SI) in favor of BCG formulation. (C) Cytotoxic T-lymphocyte (CTL) assays; mice immunized with HCVcp+F127 showed significantly higher specific lysis than mice immunized with HCVcp+BCG. Statistical analysis was done by the Mann-Whitney non-parametric test and ANOVA analyses.“*”(star labeled figure) indicates the significant differences (P < 0.05).

The frequency of HCVcp-specific spleenocytes capable of secreting IFNγ and IL4 cytokines that respectively represent activation of Th1 and Th2 immune arms (47) were measured via ex vivo ELIS pot assay. As shown in Figure 3 A, considerably higher number of SFCs that were counted for test groups (HCVcp + BCG and HCVcp+F127) verified the specific activation of cytokine-producing cells in the vaccinated animals compared to their corresponding controls (BCG and F127, respectively, both P < 0.05). In addition, HCVcp + BCG formulation promoted the generation of IFNγ-secreting cells with a significantly higher frequency compared to the HCVcp+F127 regimen (Figure 3 A) (P < 0.05), while, in accordance with the results of IgG1 subclass (Figure 2 B), HCVcp + F127 formulation indicated higher frequency of IL4-secreting cells (Figure 3 B) (P < 0.05).

As shown in Figure 3 B, assessment of the proliferation rates indicated that animal groups immunized with HCVcp + BCG had a significantly higher SI mean compared to their control counterparts and HCVcp + F127 immunized animals (P < 0.05).

Finally, to a assess the functionality of the induced cellular immune responses for either of the HCVcp formulations, we analyzed the ability of the vaccinated mice CTL reservoir for *in vivo* specific lysis of the target cells expressing the C39 epitope on their surface. This recently developed technique is now considered as a safe, easy and precise alternative for the chromium release assay that has been the classic method for quantifying cell cytotoxicity (45). Accordingly, CFSE-labeled naïve spleen cells loaded with C39 peptide were intravenously injected to the animals and were tracked by flow cytometric analysis, the following day. As shown in Fig. 3C, animals that had received the BCG or F127 adjuvants alone, had a back ground lysis of around 2-5 percent. However, the mean specific lysis in animals that were vaccinated with HCVcp immunogen was 45% and 62% for BCG and F127 adjuvant formulations, respectively, indicating the efficiency of antigen (HCVcp) formulations for specific lysis.

## 5. Discussion

In the present study and in accordance with most of the earlier studies employing BCG as an adjuvant for formulation of their antigens (34 , 48 - 50), immunization of mice by HCVcp + BCG formulation had no noticeable side effect both locally and systemically on immunized animals implying the safety of the immunogen at the administered dosage level. Our data for immunization by HCVcp+F127 were consistent with the prior report on raising HCVcp specific antibodies (Figure 2) by this formulation ( 15). But contrary to a recent report on immunization studies of *L. donovani *promastigote antigens (LAg) with BCG formulation (34), HCVcp + BCG formulation in our study elicited significant amounts of antibodies, albeit in smaller amount than that of the HCVcp+F127 formulation (Figure 2). These results are however, in agreement with the data of another recent study on immunization of mice by co-administration of killed leishmania major (KLM) vaccine and BCG on raising antibody levels comparable to that of the KLM + Alum formulation (48). Accordingly and in support of our results, a prior study had also reported on detection of signiﬁcant and speciﬁc increases in anti-IgG responses in murine model following immunization by a formulation containing BCG and FML antigen of Leishmania donovani (*L.donovani *promastigote glycoproteic complex ligand) as a candidate vaccine against visceral leishmaniasis (VL) (49).

Evaluation of cytokine levels in our study indicated higher generation of IFNγ-secreting cells for HCVcp + BCG formulation compared to that of HCVcp+F127 (Figure 3 A) (P < 0.05). These results together with data from IgG subclasses (Figure 2 A and B) clearly indicated the relatively higher Th1-oriented immune responses of the BCG adjuvant over F127 for HCVcp vaccine formulations (Table 1). Our results for superiority of BCG for induction of Th1-oriented responses were in agreement with data of KLM+BCG (48) and a TB vaccine candidate (consisted of BCG and Ag85B and ESAT-6 proteins of this bacteria) in mice immunization studies (33) and supported the cytokine results of recent studies on Lag+BCG (34) and FML+BCG (49) for production of moderate levels of IFNγ compared to other vaccine formulations.

It has been shown that formulation of BCG with other antigens is capable of induction of strong cellular responses by mechanisms such as stimulation of TLRs (28 , 29). Accordingly, immunization of mice by Lag + BCG (34) or KLM+BCG (48) induced strong delayed-type hypersensitivity (DTH; which is known as an index of cell-mediated immunity) and considerable recalls of proliferative responses in immunized animals, respectively. However, in contrast, FML-BCG immunized mice (49) failed to show any signiﬁcant footpad swellings (DTH response). Consistent with the preceding mentioned reports, our results of ex vivo recalling of *in vivo *primed spleenocytes (proliferative responses) indicated that HCVcp + BCG immunized group had a significantly higher SI mean compared to that of HCVcp+F127 formulation (Figure 3 B). This result emphasized for the dominance of BCG adjuvant compared to F127 for *in vivo *generation of HCVcp-specific lymphocytes (P < 0.05) and together with our cytokine assay results (Figure 3 A) confirmed the induction of strong and specific cellular immune responses by application of HCVcp + BCG immunogen formulation. It is also interesting to note that these results are in agreement with the data of immunization studies in rhesus macaques and a phase-I study in human for HCV core-ISCOMATRIX (formulation of HCVcp 1-191 in negatively charged immune stimulating complex) in induction of both antibody responses and T-cell cytokines in vaccines (4).

It has previously been shown that CD8+ cytotoxic T-lymphocytes were involved in the immune response against mycobacteria (31). Accordingly, a number of immunization studies have addressed the induction of antigen specific CTLs by utilization of BCG as a live vector for the expression of recombinant proteins (51) including recombinant BCG for expression of CTL epitope of HCV NS5a protein (40) and a multi-epitopic HCV construct (41). Although these recombinant BCG expressing systems provided evidence for HCV epitope-specific cellular immune responses in immunized mice yet they only carried epitopic sections of the full gene (and not the entire protein)(40 , 41). In fact, construction of recombinant BCG requires fastidious procedures with low production yields and stabilization problems which might limit their application in population scale vaccination strategies (4) while application of BCG as adjuvant to formulate other antigens would be easily available and applicable (35 -39). However, studies on application of BCG as an adjuvant to formulate other vaccine antigens for induction of CTLs are rather limited. Recently, in attempts to provide a vaccine against prostate cancer or HIV, BCG cell wall skeleton (BCG-CWS) (30) or Ag85B of mycobacteria (known as an immunogenic protein for Th1 development) (32) were shown to enhance the induction of CTL responses against prostate-specific antigen (PSA) or HIV gp120, respectively. More recently, presence of BCG-Hsp65 in a HBV CD8+ CTL epitopic vaccine was shown to enhance induction of HBV specific CTLs (52). Consistent with these prior reports, our results for assessment of the CTL responses, indicated 45% mean specific lysis in HCVcp + BCG immunized mice (Figure 3 C). These results indicated moderate CTL responses for HCVcp + BCG formulation and in accordance with cytokine (Figure 3 A) and proliferation data (Figure 3 B) onfirmed the effectiveness of BCG adjuvant for induction of cellular responses by HCVcp immunization. A point of discrepancy in our results however was observation of 62% mean specific lysis in case ofHCVcp+F127 immunized mice (Figure 3 C) which is in contrary with the results of a prior study (15) reporting the ineffectiveness of HCVcp + F127 formulation in induction of CTLs (especially compared to HCVcp + Montanide ISA720 formulation). The reason behind this discrepancy might be the employed method of CTL assessment in the prior study (lactate dehydrogenase (LDH) assay) which was a rather old and not precise method compared to our CFSE dye assay which is now considered as a proper alternate for the chromium release method that is the standard assay for quantifying CTLs (45, 53). In fact, besides activation of humoral responses, F127 has also been proposed to induce CTL responses through mechanisms that involve the production of micelles that facilitate the non-specific transfer of protein antigens into the cytoplasm as was shown for induction of virus-specific CD8+ CTL responses against lymphocytic choriomeningitis virus infection (54, 55). These propositions are in accordance with our results for the observed IgG and CTL responses for HCVcp-F127 formulations while our observation of moderate CTL responses in HCVcp + BCG immunized mice is also consistent with results of a recent study on superiority KLM-Montandide ISA720 compared to KLM-BCG formulation (48) in induction of antigen specific cellular immune responses.

Taken together, the present study is the first (to the best of our knowledge) to address immunization studies on formulation of HCV proteins with BCG as an adjuvant system. To conduct a comparative analysis, we evaluated both HCVcp + BCG and HCV + F127 formulations in separate groups of immunized mice. Our results indicated that HCVcp + BCG induced a moderate CTL and mixed Th1/Th2 (with higher Th1 switching) with higher levels of cell proliferation and IFN- γ secretion compared to that of the HCVcp + F127 formulation indicating that BCG may have a better outcome when formulated in HCVcp-based subunit vaccines.
